# Low Bone Turnover Due to Hypothyroidism or Anti-Resorptive Treatment Does Not Affect Whole-Body Glucose Homeostasis in Male Mice

**DOI:** 10.3390/jpm12091462

**Published:** 2022-09-06

**Authors:** Franziska Lademann, Martina Rauner, Nicolas Bonnet, Lorenz C. Hofbauer, Elena Tsourdi

**Affiliations:** 1 Department of Medicine III and Center for Healthy Aging, Technical University Dresden Medical Center, 01307 Dresden, Germany; 2 Division of Bone Diseases, Department of Internal Medicine Specialties, Geneva University Hospital & Faculty of Medicine, 1211 Geneva, Switzerland

**Keywords:** bone metabolism, glucose homeostasis, glucose uptake, thyroid hormones, hypothyroidism, bisphosphonates

## Abstract

Bone is a large and dynamic tissue and its maintenance requires high amounts of energy as old or damaged bone structures need to be replaced during the process of bone remodeling. Glucose homeostasis is an essential prerequisite for a healthy bone and vice versa, the skeleton can act as an endocrine organ on energy metabolism. We recently showed that hypothyroidism in mice leads to an almost complete arrest of bone remodeling. Here, we aimed to investigate whether the profound suppression of bone remodeling affects whole-body glucose homeostasis. To that end, male C57BL/6JRj mice were rendered hypothyroid over 4 weeks using methimazole and sodium perchlorate in the drinking water. We confirmed trabecular bone gain due to decreased bone turnover in hypothyroid mice with decreased cortical but increased vertebral bone strength. Further, we found impaired glucose handling but not insulin resistance with hypothyroidism. In hypothyroid bone, glucose uptake and expression of glucose transporter *Glut4* were reduced by 44.3% and 13.9%, respectively, suggesting lower energy demands. Nevertheless, hypothyroidism led to distinct changes in glucose uptake in muscle, liver, and epididymal white adipose tissue (eWAT). Reduced glucose uptake (−30.6%) and *Glut1*/*Glut4* transcript levels (−31.9%/−67.5%) were detected in muscle tissue. In contrast, in liver and eWAT we observed increased glucose uptake by 25.6% and 68.6%, respectively, and upregulated expression of glucose transporters with hypothyroidism. To more specifically target bone metabolism and discriminate between the skeletal and systemic effects of hypothyroidism on energy metabolism, male mice were treated with zoledronate (ZOL), a bisphosphonate, that led to decreased bone turnover, trabecular bone gain, and reduced local glucose uptake into bone (−40.4%). However, ZOL-treated mice did not display alterations of systemic glucose handling nor insulin tolerance. Despite the close mutual crosstalk of bone and glucose metabolism, in this study, we show that suppressing bone remodeling does not influence whole-body glucose homeostasis in male mice.

## 1. Introduction

Bone is a living dynamic tissue and the constant process of bone remodeling requires high amounts of glucose as the main energy source of both bone-forming osteoblasts and bone-resorbing osteoclasts [1,2]. The balance of bone formation and resorption is a prerequisite for bone homeostasis and thus, a healthy bone. In response to extrinsic and intrinsic factors such as mechanical stress, nutrients, and hormones, coordinated actions of osteoclasts and osteoblasts under the direction of regulatory osteocytes help to adapt bone mass and shape accordingly. Thyroid hormones 3,3′,5-triiodo-l-thyronine (T3) and l-thyroxine (T4) are important regulators of energy metabolism in general, skeletal development, and bone maintenance. Thyroid disorders such as hypothyroidism are common and affect both bone and glucose metabolism [3,4]. Hypothyroid patients present with wide variety of symptoms such as fatigue, weight gain, cognitive impairment, mood fluctuations, and cold intolerance, which can differ with age, sex, time between onset and diagnosis, and severity of hypothyroidism [4]. Thyroid hormone deficiency has clinical implications to almost all major organs and can also lead to disturbances of glucose metabolism and a low metabolic rate [4]. In line, hypothyroid adults show a prolonged bone remodeling cycle leading to low bone turnover and increased secondary mineralization [5,6,7]. Additionally, mouse models have been established to study the effects of thyroid hormone deficiency on metabolism. Hypothyroid mice have a lean phenotype despite reduced heart rate, low energy expenditure, and decreased locomotor activity [8,9]. Further, they show white adipose tissue (WAT) browning [10] and can develop nonalcoholic fatty liver disease [11]. In our previous study, we reported trabecular bone gain due to hypothyroidism in male mice, resulting from reduced bone turnover and a prolongated bone remodeling cycle [12]. In line, we observed decreased bone remodeling with increased bone mass in mice treated with the bisphosphonate zoledronate [13], however, in both cases we did not assess whether a low bone turnover might also lead to reduced glucose uptake and metabolism in bone with possible systemic consequences.

Lately, the skeleton has emerged as an endocrine organ secreting osteokines such as osteocalcin (OCN), receptor activator of NF-κB ligand (RANKL) and sclerostin (SOST) that can regulate systemic glucose homeostasis [14,15,16,17]. Daily injections of uncarboxylated osteocalcin improve glucose tolerance and insulin tolerance in mice fed a normal diet and can prevent the development of type 2 diabetes mellitus in mice on high-fat diet [18]. Based on studies in bone-specific knockout mice, increased insulin signaling in osteoblasts promoted whole-body glucose metabolism by enhancing bone resorption and activation of osteocalcin [19,20]. RANKL, besides its major role in osteoclast differentiation and activation, can regulate insulin sensitivity [21] and beige adipocyte differentiation from preadipocytes, thereby controlling energy expenditure in mice [22]. Furthermore, *Sost* knockout mice exhibit not only osteosclerotic bones, but also reduced adipogenesis and increased insulin sensitivity, while *Sost* overexpression led to opposite metabolic consequences characterized by adipocyte hyperthrophy [23]. These findings support a role of bone tissue secreted factors in systemic glucose handling.

In this study, we aimed to determine whether the arrested bone turnover through pharmacologically induced hypothyroidism or treatment with zoledronate might affect whole-body glucose homeostasis in mice.

## 2. Methods

### 2.1. Animal Experiments

Animal procedures were approved by the Institutional Animal Care Committee of the Technische Universität Dresden and the Landesdirektion Sachsen (TVV 2015/03). Twelve-week-old male C57BL/6JRj mice were purchased from Janvier Labs and housed under institutional guidelines. Animals were maintained in groups up to 4 animals in a light–dark cycle of 12/12 h at room temperature in filter-top cages and had ad libitum access to their respective drinking water and standard chow diet.

In the first experimental setup, mice were rendered hypothyroid by adding 1% (wt/vol) sodium perchlorate and 0.1% (wt/vol) methimazole (both Sigma-Aldrich Chemie GmbH, Taufkirchen, Germany) to the drinking water over 4 weeks (HYPO), as described previously [12]. Euthyroid mice received normal drinking water (CO). Our established protocol leads to a severe reduction in total T3 and total T4 levels, as reported previously [12].

In our second trial, mice were injected intraperitoneally with 100 mg/kg zoledronate (ZOL) (Sigma-Aldrich) or phosphate-buffered saline as vehicle control (PBS) once per week over 4 weeks.

After 21 days, animals were subjected to intraperitoneal glucose injections to assess their glucose tolerance (ipGTT). After 16 h of fasting, glucose levels were measured in tail vein blood using a glucometer (ACCU CHEK Aviva III, Roche Diabetes Care, Mannheim, Germany). Subsequently, animals received intraperitoneal injections of 2 g/kg body weight D-glucose (Sigma-Aldrich) and blood glucose levels were tested after 15, 30, 60, 90, and 120 min. Four days after the ipGTT, an intraperitoneal insulin tolerance test (ipITT) was performed. First, mice were fasted over 4 h prior to the test and initial blood glucose concentration was quantified using tail vein blood. Mice received intraperitoneal injections of 0.4 IE/kg body weight insulin (Huminsulin^®^, 100 IE/mL, Lilly Deutschland GmbH, Bad Homburg, Germany) and blood glucose levels were measured after 15, 30, 60, 90, 120, and 180 min. During ipGTT and ipITT, all mice had ad libitum access to their respective drinking water. The area under the curve (AUC) was generated to compare the glucose clearance from the blood over time among the experimental groups.

Four weeks after starting the treatments, mice were euthanized using CO_2_. Blood was collected via heart puncture and serum was obtained by centrifugation. For subsequent bone analyses, fourth lumbar vertebrae (L4) and femurs were collected postmortem, fixed in 4% PBS-buffered paraformaldehyde for 48 h, and stored in 50% ethanol. Furthermore, tissue of different organs (tibia, muscle gastrocnemius, liver, subcutaneous and epididymal white adipose tissue (sWAT and eWAT), brown adipose tissue (BAT)) and bone marrow were collected and cryoconserved for subsequent analysis.

A separate set of mice was used for 2-[14C]-Deoxy-Glucose (2-14C-DG) uptake assay. For local glucose uptake measurements in tibia, muscle, eWAT and liver, a bolus of 2-deoxy-d-[1-14C] glucose (15μCi, PerkinElmer, Waltham, MA, USA) was injected into the tail vein of mice that were fasted beforehand over 3 h. After 30 min, mice were sacrificed and tissues were rapidly collected, frozen in liquid nitrogen, and stored at −80 °C for subsequent analysis. The amount of 2-14C-DG in total tissue lysates was quantified by a liquid scintillation counter (Wallac 1414 WinSpectral v1.40 Scilliation Counter, Perkin Elmer) and normalized to the tissue weight.

### 2.2. Serum Analysis

Serum concentrations of bone turnover markers procollagen type 1 amino-terminal propeptide (P1NP) and tartrate-resistant acid phosphatase (TRAP) were measured using ELISAs according to the manufacturer’s protocol (AC-33F1 and SB-TR103, both IDS, Frankfurt am Main, Germany). Serum levels of creatine kinase, cholesterol, HDL-cholesterol, LDL-cholesterol, triglycerides, and free fatty acids were assessed using the Cobas 8000 modular analyzer (Roche, Mannheim, Germany) and the respective test kits (CK: REF 05,168,546 from Roche, CHOL2: REF05168538 from Roche, HDLC4: REF0758582 from Roche, LDLC3: REF07005768 from Roche, TRIGL: REF05171407 from Roche; NEFA-HR(2) REF434-91795 from Wako Chemicals GmbH, Neuss, Germany).

### 2.3. Analysis of Bone Mass, Microarchitecture, and Strength

To determine bone mass and microarchitecture, the distal femur and fourth lumbar vertebra (L4) were analyzed using microcomputed tomography (micro-CT) (vivaCT40, Scanco Medical, Brüttisellen, Switzerland) with an X-ray energy of 70 kVp and isotropic voxel size of 10.5 μm (114 mA, 200 ms integration time). Trabecular (Tb.) and cortical (Ct.) bone parameters including bone volume/total volume (BV/TV), trabecular number (Tb.N), trabecular separation (Tb.Sp), and thickness (Tb.Th) were assessed based on calculations including 100 scan slices following standard protocols from Scanco Medical. Trabecular bone parameters of femurs were assessed in the metaphyseal region starting 20 slices below the growth plate, while cortical bone analysis was performed within the diaphyseal region midway between femoral head and distal condyles. Trabecular bone of L4 was evaluated at the center contouring 50 slices above and 50 slices below the middle of the vertebral body.

Femurs were used for 3-point bend testing to assess cortical bone strength while L5 vertebrae were used for a compression test (Zwick Roell, Ulm, Germany). Therefore, femurs and L5 vertebrae were rehydrated in PBS overnight. Femurs were placed onto two supports with an intermediate distance of 6 mm, while vertebrae were placed onto the center of the lower plate. Mechanical force was applied vertically onto the middle of the femoral midshaft and onto L5 vertebra via the upper platen, respectively. After reaching a preload of 1 N, the measurement started and continued with a load rate of 0.05 mm/s until failure. The maximal load (F_max_) and elastic modulus (E_mod_) were quantified as an indicator of bone strength and stiffness, respectively, using testXpert II—V3.7 software (Zwick Roell, Ulm, Germany).

### 2.4. Osmium Staining

For the quantification of bone marrow fat content (MAT), intact fixed femurs were decalcified (Osteosoft^®^, Merck, Kenilworth, NJ, USA) for one week. After scanning the femurs with the micro-CT to ensure complete decalcification, bones were briefly washed in PBS and stained for 1 h with 2% osmium tetroxide (Electron Microscopy Science, Hatfield, PA, USA) to label fat droplets within the bone marrow adipocytes [24]. Femurs were transferred into PBS, scanned (10.5 μm voxel size, 300 ms integration time, 70 kVP) and evaluated regarding their bone marrow fat content by using the vivaCT40.

### 2.5. Histology

Liver tissue, sWAT, eWAT, and BAT were fixed in 4% PBS-buffered paraformaldehyde, dehydrated using an ascending ethanol series, and embedded in paraffin. Sections (4 μm) were prepared and stained with hematoxylin/eosin (HE) to visualize tissue structures. Representative photos were taken using Microscope Axio Imager M1, (Carl Zeiss Jena, Jena, Germany) and CellSens Entry Software Version 1.5 (OLYMPUS Cooperation, Shinjuku, Japan).

### 2.6. RNA Isolation, RT-PCR, and Quantitative Real-Time PCR

Total RNA from tissues and bone marrow was extracted using TRIzol reagent (Invitrogen, Darmstadt, Germany) following the manufacturer’s protocol and quantified using a Nanodrop spectrophotometer (Peqlab, Erlangen, Germany).

Five hundred nanograms of RNA were reverse transcribed using Superscript II (Invitrogen, Darmstadt, Germany) followed by SYBR Green-based quantitative real-time PCR according to established protocols (ABI7500 Fast, Applied Biosystems, Carlsbad, CA, USA). Primer sequences are listed in Supplemental Table S1. PCR conditions were: 50 °C for 5 min and 95 °C for 10 min followed by 40 cycles with 95 °C for 15 s and 60 °C for 1 min. Melting curves were evaluated using the following scheme: 95 °C for 15 s, 60 °C for 1 min and 95 °C for 30 s. Results were calculated based on the ∆∆CT method and are represented as x-fold increase normalized to *β-actin, Tbp*, or *Hprt1* mRNA levels.

### 2.7. Statistical Analysis

Data are presented as mean ± 95% confidence interval (CI). Each dot indicates an individual mouse. Statistical analysis comparing two groups are based on a two-sided unpaired Student’s *t*-test using GraphPad Prism 7.0 (GraphPad, La Jolla, CA, USA). Values of *p* < 0.05 were considered statistically significant. Significant outliers were excluded based on Grubbs’ test provided in GraphPad by Dotmatics. (https://www.graphpad.com/quickcalcs/grubbs1/ (accessed on 2 July 2022)).

## 3. Results

### 3.1. Hypothyroidism Leads to Low Bone Turnover and Increases Trabecular Bone Mass

**Figure 1:**
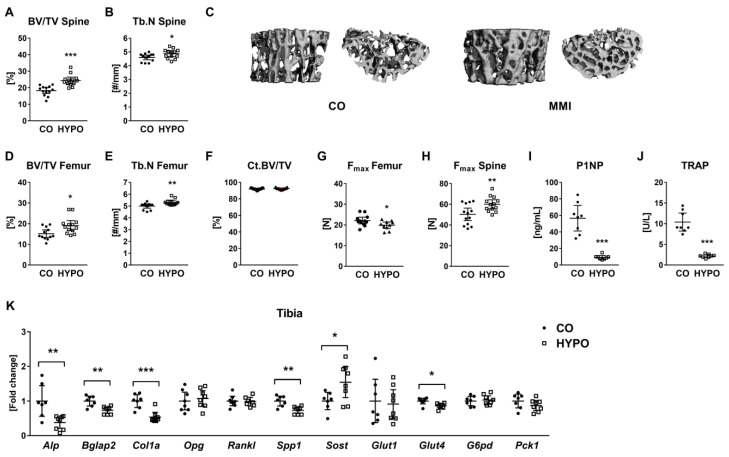
Experimental hypothyroidism leads to trabecular bone gain and reduced bone turnover in male mice. Bones from 16-week-old male hypothyroid (HYPO) and control (CO) mice were examined by microCT. At the spine, (**A**) bone volume/total volume (BV/TV) and (**B**) trabecular number (Tb.N) were determined. (**C**) Representative 3D reconstructions of the trabecular compartment of L4 vertebrae from hypothyroid and control mice. Additionally, (**D**) BV/TV and (**E**) Tb.N were assessed in the distal femur. (**F**) Cortical bone volume/total volume (Ct.BV/TV) was measured at the femoral midshaft. As an indicator of cortical bone strength, (**G**) maximal force (F_max_) was determined by 3-point bending test of femurs. (**H**) Vertebral bone strength indicated by F_max_ was determined by a compression test of the L5 vertebra. Furthermore, serum concentrations of (**I**) bone formation marker P1NP and (**J**) bone resorption marker TRAP were measured using ELISAs. (**K**) mRNA expression of *Alp*, *Bglap2*, *Col1a*, *Opg*, *Rankl*, *Spp1*, *Sost*, glucose transporter *Glut1* and *Glut4*, and *G6pd* and *Pck1* in tibia bone tissue obtained from control and hypothyroid mice was quantified by real-time PCR. Each dot indicates an individual mouse. The horizontal lines represent the mean ± 95% CI. MicroCT analysis: N = 13 per group; biomechanical testing: CO: N = 12, HYPO: N = 10; ELISA: N = 8 per group and real-time PCR: CO: N = 7, HYPO: N = 8. Statistical analysis was performed by Student’s *t*-test: * *p* < 0.05; ** *p* < 0.01; *** *p* < 0.001 vs. control.

In accordance with our previous study [12], we observed trabecular bone gain in hypothyroid mice (Figure 1 and Figure S1) as exemplarily shown by increased bone volume over total volume and trabecular numbers at the spine (BV/TV: +33.31%; Tb.N: +5.85%; Figure 1A–C) and femur (BV/TV: +26.07%; Tb.N: +6.35%, Figure 1C,D). Cortical bone structure was not altered (Figure 1F and Figure S1H,I); however, three-point bending of femurs indicated reduced cortical bone strength and elastic modulus in hypothyroid mice (F_max_: −10.16%, E_mod_: −17.91%, Figure 1G and Figure S1J). In contrast, L4 vertebra of hypothyroid animals showed an increase in F_max_ and E_mod_ by 1.19-fold and 1.37-fold (Figure 1H and Figure S1D), respectively, during compression test. Serum analysis of bone turnover markers revealed reduced levels of bone formation marker P1NP and bone resorption marker TRAP, substantiating bone remodeling arrest due to hypothyroidism (Figure 1I,J). In bone tissue of hypothyroid mice, we further detected reduced mRNA transcript levels of typical osteoblast marker genes (*Alp*: −62.22%, *Bglap2*: −26.64%, *Col1a*: −46.47%, *Spp1*: −26.63%), while *Rankl* and *Opg* expression remained unchanged (Figure 1K). Expression of osteocyte-derived *Sost* was upregulated 1.54-fold in hypothyroidism, as described previously [12] (Figure 1K). Concerning glucose metabolism, we detected decreased mRNA levels of *Glut4* (−13.87%), but unaltered expression of *G6pd*, the rate-limiting enzyme of the pentose phosphate pathway, *Glut1* and *Pck1* that catalyzes a rate-controlling step of gluconeogenesis (Figure 1K).

### 3.2. Whole-Body Glucose Metabolism and Tissue-Specific Glucose Uptake Are Altered in Hypothyroid Mice

**Figure 2:**
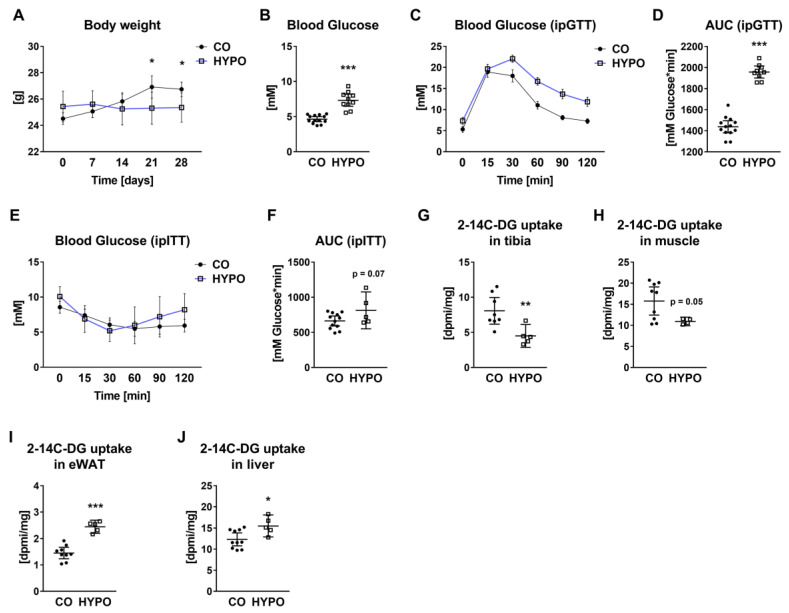
Glucose tolerance, but not insulin sensitivity is impaired in hypothyroid mice. (**A**) Body weights of hypothyroid (HYPO) and control (CO) mice were monitored throughout the 4-week experiment. (**B**) After 16 h of fasting, blood glucose levels were determined from tail vein blood prior to intraperitoneal glucose tolerance test (ipGTT). (**C**) ipGTT started with ip injection of a 20% *v/v* glucose solution. Blood glucose concentrations were quantified at indicated times up to 2 h. (**D**) The area under the curve (AUC) was calculated to compare glucose tolerance of hypothyroid and control mice. (**E**) Furthermore, intraperitoneal insulin tolerance testing (ipITT) was performed and (**F**) AUC was calculated based on blood glucose levels over time. 2-[14C]-Deoxy-Glucose (2-14C-DG) uptake was tested in (**G**) tibia, (**H**) muscle, (**I**) epididymal WAT (eWAT), and (**J**) liver of hypothyroid vs. control mice. Each dot indicates an individual mouse. The horizontal lines represent the mean ± 95% CI. Body weight curves, fasting blood glucose, and ipGTT: CO: N = 13, HYPO: N = 10; ipITT: CO: N = 12, HYPO: N = 5; 2-14C-DG uptake: CO: N = 10, HYPO: N = 5. Statistical analysis was performed by Student’s *t*-test: * *p* < 0.05; ** *p* < 0.01; *** *p* < 0.001 vs. control.

In contrast to the symptoms reported in humans [4], hypothyroid mice displayed significant weight loss 21 and 28 days after starting the methimazole/sodium perchlorate treatment (21 days: −5.98%, 28 days: −5.21%; Figure 2A) accompanied by 1.57-fold elevated fasting blood glucose levels at day 21 (Figure 2B). Intraperitoneal blood glucose tolerance testing (ipGTT) indicated impaired glucose tolerance in hypothyroid mice as shown by a 36.16% elevated area under the curve (Figure 2C,D). Insulin tolerance was not significantly affected by hypothyroidism (Figure 2E,F). Using a 2-[14C]-Deoxy-Glucose (2-14C-DG) uptake assay, we detected reduced local 2-14C-DG uptake into bone (−44.28%) and muscles (−30.57%), while elevated uptake into eWAT (+68.59%) and liver tissue (+25.62%) (Figure 2G–J) of hypothyroid animals.

### 3.3. Muscle, Liver, and Adipose Tissues Are Distinctly Affected by Hypothyroidism

**Figure 3:**
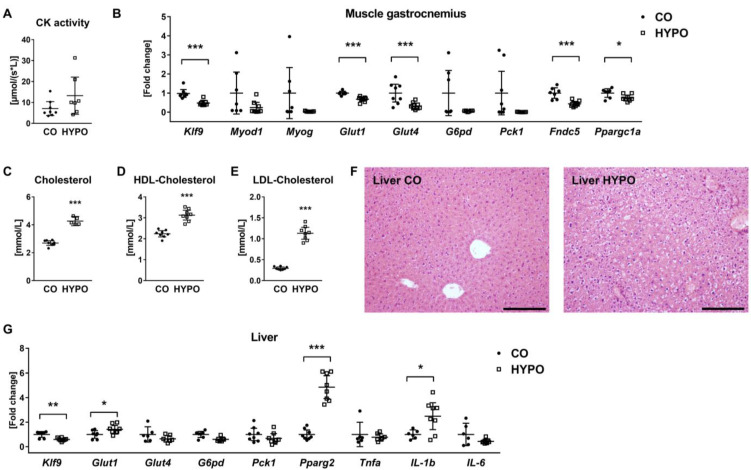
Hypothyroid mice display reduced mRNA expression of glucose transporters *Glut1* and *Glut4* in muscle together with high serum cholesterol and pathological changes in liver tissue. Muscle, serum, and liver tissue from 16-week-old male hypothyroid (HYPO) and control (CO) mice were collected and tested using Cobas 8000 modular analyzer, real-time PCR analysis, and histology. (**A**) Creatine kinase serum levels and (**B**) mRNA expression of *Klf9*, myoblast differentiation marker genes *MyoD1* and *Myog*, glucose transporters *Glut1* and *Glut4*, *G6pd*, *Pck1*, *Fndc5*, the precursor for irisin, and its transcriptional regulator *Pppargc1a* in muscle tissue are compared between control and hypothyroid mice. Further, serum levels of (**C**) cholesterol, (**D**) HDL-cholesterol, and (**E**) LDL-cholesterol were determined. (**F**) Representative pictures of HE-stained liver sections. (**G**) mRNA expression of *Klf9*, *Glut1*, *Glut4*, *G6pd*, adipocyte marker *Ppparg2*, and proinflammatory cytokines *Tnfa*, *IL-1b*, and *IL-6* is shown in liver tissue for control versus hypothyroid mice. Each dot indicates an individual mouse. The horizontal lines represent the mean ± 95% CI. Serum analyses: N = 8 per group; real-time PCR: CO: N = 7, HYPO: N = 8. Statistical analysis was performed by Student’s *t*-test: * *p* < 0.05; ** *p* < 0.01; *** *p* < 0.001 vs. control.

As whole-body glucose homeostasis was affected by hypothyroidism, yet, we observed an organ-specific 2-14C-DG uptake in hypothyroid mice, we further characterized serum parameters, histology, and mRNA expression profiles related to tissues potentially contributing to glucose homeostasis. Concerning muscles, creatine kinase serum levels tended to increase by 1.87-fold with hypothyroidism (*p* = 0.12, Figure 3A). RNA expression analysis revealed downregulated levels of thyroid hormone target gene *Klf9* transcripts by 52.01%, indicating local hypothyroidism, in muscle tissue, while myogenic marker genes *MyoD1* and *Myog* were not affected by hypothyroidism (Figure 3B). Further, we detected reduced expression of glucose transporters *Glut1* and *Glut4* by 31.87% and 67.51%, respectively, while transcript levels of *G6pd* and *Pck1* were not significantly altered (Figure 3B). Interestingly, expression of *Fndc5* coding for the myokine irisin and its transcriptional regulator *Ppargc1a* was decreased by 56.2% and 25.35%, respectively, in hypothyroid mice (Figure 3B).

In the serum of hypothyroid animals, we found increased levels of total cholesterol by 1.58-fold, HDL-cholesterol by 1.39-fold, and LDL-cholesterol by 3.83-fold (Figure 3C,D). Histological analysis revealed an abnormal liver structure including ballooning degeneration of hepatocytes and vascular congestion (Figure 3F). At the transcriptional level, expression of *Klf9* decreased by 40.83%, while expression of *Glut1*, *Pparg*, and *Il-1b* increased due to hypothyroidism (Figure 3G). Transcript levels of *Glut4*, *G6pd, Pck1*, *Tnfa*, and *Il-6* remained unchanged (Figure 3G).

**Figure 4:**
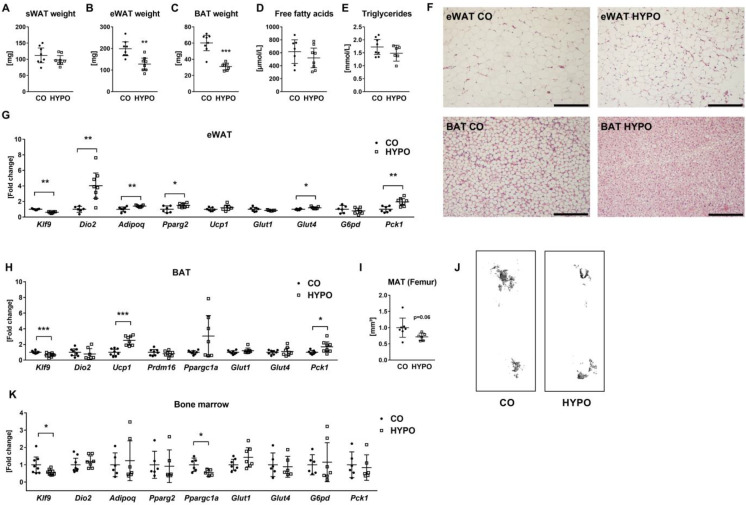
Lack of thyroid hormones results in reduced eWAT and BAT weights while increased white and brown adipocyte marker expression, respectively. Subcutaneous white adipose tissue (sWAT), epididymal white adipose tissue (eWAT), brown adipose tissue (BAT), and serum from 16-week-old male hypothyroid (HYPO) and control (CO) mice were collected and tested using Cobas 8000 modular analyzer, real-time PCR analysis, and histology. (**A**) sWAT, (**B**) eWAT, and (**C**) BAT were weighted postmortem. Levels of (**D**) free fatty acids and (**E**) triglycerides were measured in the serum. (**F**) Representative pictures of HE-stained eWAT and BAT sections from control and hypothyroid mice. Furthermore, (**G**) mRNA expression of *Klf9*, *Dio2*, adipocyte marker *Adipoq* and *Pparg2*, and brown adipocyte marker *Ucp1*, together with *Glut1*, *Glut4*, *G6pd*, and *Pck1* was quantified with real-time PCR analysis in eWAT. (**H**) Transcript levels of *Klf9*, *Dio2*, *Ucp1*, *Prdm16*, *Ppargc1a*, *Glut1*, *Glut4*, and *Pck1* were assessed in BAT from control and hypothyroid mice. Femurs from 16-week-old male hypothyroid and control mice were examined after osmium staining using microCT and (**I**) bone marrow adipose tissue volume (MAT) was quantified. (**J**) Representative pictures showing 3D reconstructions of MAT within femurs of control and hypothyroid mice. (**K**) mRNA expression of *Klf9*, *Dio2*, *Adipoq*, *Pparg2*, *Ppargc1a*, *Glut1*, *Glut4*, *G6pd*, and *Pck1* was measured using real-time PCR. Each dot indicates an individual mouse. The horizontal lines represent the mean ± 95% CI. Tissue weights and serum analyses: CO: N = 8, HYPO: N = 7; real-time PCR: CO: N = 7, HYPO: N = 8; Osmium staining: CO: N = 7, HYPO: N = 6. Statistical analysis was performed by Student’s *t*-test: * *p* < 0.05; ** *p* < 0.01; *** *p* < 0.001 vs. control.

Regarding adipose tissue, we observed unchanged weights of subcutaneous WAT (sWAT), but loss of epididymal WAT (eWAT) and brown adipose tissue (BAT) by 35.55% and 48.44%, respectively, (Figure 4A–C) in hypothyroid animals. In line, adipose tissue histology revealed reduced adipocyte size in eWAT and BAT in hypothyroid mice (Figure 4F); however, serum concentrations of free fatty acids and triglycerides were not significantly affected (Figure 4D,E). Within eWAT, low expression of *Klf9* while high expression of deiodinase 2 (*Dio2*), an enzyme that can convert T4 to T3, indicated local hypothyroidism in treated mice (Figure 4G). In contrast to histology, adipocyte marker genes *Adipoq* and *Pparg2* were upregulated by 40.69% and 49.36%, respectively, and enhanced expression of *Glut4* (+18.88%) and *Pck1* (+95.18%) suggest increased glucose demand in hypothyroid adipocytes (Figure 4G). In BAT, we found reduced *Klf9* (−34.82%) expression and 2.51-fold increased mRNA levels of *Ucp1*, the master regulator of brown adipocyte thermogenesis, although *Prdm16*, the transcriptional coregulator of brown adipocyte differentiation, and *Ppargc1a* were not regulated by hypothyroidism (Figure 4H). While expression of glucose transporters remained unchanged, 1.7-fold increased *Pck1* transcript levels indicated upregulated gluconeogenesis in BAT lacking thyroid hormones (Figure 4H).

Using osmium staining, we detected a tendency for decreased bone marrow fat content inside femurs of hypothyroid animals (−28.41%, *p* = 0.06) and downregulated expression of *Klf9* and *Ppargc1a*, while transcript levels of the other selected genes were not altered, including glucose transporters and genes involved in glucose metabolism (Figure 4I–K).

Overall, we found tissue-specific effects of hypothyroidism on local glucose metabolism either affecting glucose uptake, gluconeogenesis, and/or cellular activity, suggesting that impaired glucose metabolism in hypothyroidism may not only be attributed to the reduction in bone turnover.

### 3.4. Bisphosphonate Treatment Impairs Bone Turnover, but Not Systemic Glucose Homeostasis

**Figure 5:**
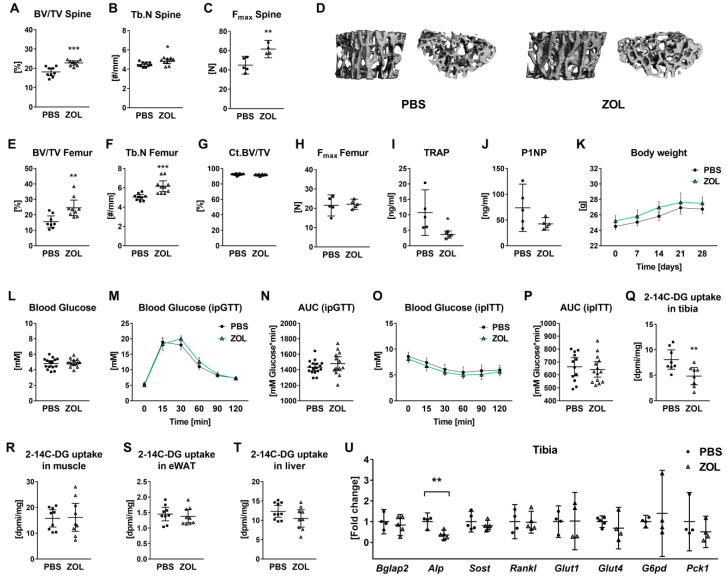
Zoledronate arrests bone resorption but does not affect whole-body glucose metabolism in male mice. Bones from 16-week-old male control mice (CO) and mice treated with zoledronate (ZOL) were tested by microCT. At the spine, (**A**) bone volume/total volume (BV/TV) and (**B**) trabecular number (Tb.N) were assessed. (**C**) Vertebral bone strength indicated by F_max_ was determined by a compression test of the L5 vertebra. (**D**) Representative 3D reconstructions of the trabecular compartment of L4 vertebrae from ZOL-treated and control mice. Furthermore, (**E**) BV/TV and (**F**) Tb.N were measured in the distal femur. (**G**) Cortical bone volume/total volume (Ct.BV/TV) was determined at the femoral midshaft. As an indicator of cortical bone strength, (**H**) maximal force (F_max_) was quantified by 3-point bending test of femurs. In addition, serum concentrations of (**I**) bone formation marker P1NP and (**J**) bone resorption marker TRAP were measured using ELISAs. (**K**) Body weights were monitored throughout the 4-week experiment. (**L**) After 16 h of fasting, blood glucose levels were assessed from tail vein blood prior to intraperitoneal glucose tolerance test (ipGTT). (**M**) ipGTT started with ip injection of a 20% *v/v* glucose solution. Blood glucose concentrations were quantified at indicated times up to 2 h and (**N**) the area under the curve (AUC) was calculated to compare glucose tolerance of hypothyroid and control mice. (**O**) Furthermore, intraperitoneal insulin tolerance testing (ipITT) was performed and (**P**) AUC was calculated based on blood glucose levels over time. 2-[14C]-Deoxy-Glucose (2-14C-DG) uptake was determined in (**Q**) tibia, (**R**) muscle, (**S**) epididymal WAT (eWAT), and (**T**) liver. (**U**) mRNA expression of *Bglap2*, *Alp*, *Sost*, *Glut1*, *Glut4*, *G6pd*, and *Pck1* in tibia bone tissue obtained from control and ZOL-treated mice was quantified by real-time PCR. Each dot indicates an individual mouse. The horizontal lines represent the mean ± 95% CI. MicroCT analysis: N = 10 per group; biomechanical testing: N = 5 per group; ELISA: N = 5 per group; real-time PCR: N = 5 per group; body weight curves: N = 16 per group; fasting blood glucose: N = 15 per group; ipGTT: PBS: N = 16, ZOL: N = 13; ipITT: PBS: N = 12, ZOL: N = 14; 2-14C-DG uptake: PBS: N = 10, ZOL: N = 9. Statistical analysis was performed by Student’s *t*-test: * *p* < 0.05; ** *p* < 0.01; *** *p* < 0.001 vs. control.

Given that hypothyroidism is a multiorgan disease that makes it difficult to draw conclusions about the effects of reduced glucose uptake by bone on whole body glucose handling, we choose the bisphosphonate zoledronate (ZOL) as a systemic treatment that decelerates bone metabolism, as described before without any major effects on other organs [13]. ZOL-treated mice were characterized by trabecular bone gain and enhanced bone strength exemplarily shown by increased bone volume (+25.68%), trabecular number (+7.53%), and maximum load (+37.1%) using microCT analysis and a compression test, respectively (Figure 5A–D and Figure S2A–D). Further, femoral trabecular bone volume and trabecular number were elevated with ZOL, while cortical bone parameters and cortical bone strength were not affected (Figure 5E–H and Figure S2E–J). Reduced serum levels of TRAP confirmed the anti-resorptive effects of ZOL (−66.33%, Figure 5I). The bone formation marker P1NP showed a tendency to decrease in serum of ZOL-treated animals (−42.27%, *p* = 0.106, Figure 5J). Nevertheless, ZOL treatment did not affect body weight, fasting blood glucose levels, glucose tolerance, or insulin sensitivity in mice (Figure 5K–P). In accordance with reduced bone metabolism, 2-14C-DG uptake was diminished in ZOL-treated mice (−40.41%, Figure 5Q), but not muscles, eWAT, and liver tissue (Figure 5R–T). Expression of *Alp*, but not *Bglap2, Rankl*, and *Sost*, was downregulated by 48.7% with ZOL in bone tissue (Figure 5U). Transcript levels of genes involved in glucose uptake and metabolism were not significantly affected in ZOL-treated mice (Figure 5U).

## 4. Discussion

Bone remodeling, the essential process to renew bone and maintain a healthy bone structure, requires large amounts of energy, mainly in the form of glucose. As our previous studies indicated profound suppression of bone turnover under hypothyroid conditions, we asked whether this would also translate into altered whole-body glucose homeostasis.

As reported previously, severely hypothyroid mice displayed increased trabecular bone mass due to reduced bone turnover [12,25]. In line with low bone turnover activity, cortical bone strength at the femur and thus likely bone quality were impaired with hypothyroidism compliant with increased bone fragility seen in *Dio2*-deficient mice with a local hypothyroid state in bone [26]. In contrast, vertebrae did not exhibit increased fragility in hypothyroid mice, likely due to net trabecular bone gain. In line, clinical studies link overt hypothyroidism in patients to increased fracture risk, especially in femur and humerus [27,28,29,30,31].

As expected, the low bone turnover in hypothyroid mice led to a decreased skeletal glucose uptake and expression of *Glut4*, corroborating lower energy demands with low metabolic activity in bone. In addition, besides the loss of body weight in hypothyroid mice, which is in agreement with studies using other either genetically modified or drug-induced mouse models of hypothyrodisim [8,10,11], we found impaired glucose handling but not insulin sensitivity in hypothyroid mice, suggesting that low bone turnover could impact whole-body glucose homeostasis. However, considering that hypothyroidism is a multiorgan disease with a low metabolic rate in general, effects on glucose metabolism can be ascribed to changes in multiple tissues [32]. Therefore, we also assessed the function and glucose uptake in other tissues associated with glucose handling. In muscle tissue, we found a reduced glucose uptake and also downregulation of genes involved in glucose uptake with hypothyroidism, while CK levels in serum as a measure of muscle exertion tended to increase. In clinical studies, an inverse relation of serum levels of T3 and CK has already been established as hypothyroid muscles become weaker and exertion leads to muscle cell damage and thus leakage of CK into the circulation [33]. A recent study by Kaspari et al. linked the lean phenotype of transgenic mice displaying hypothyroidism to increased adaptive thermogenesis in skeletal muscles as an attempt to maintain body temperature [8]. Further, this upregulated adaptive thermogenesis was associated with increased fatty acid oxidation [8] implying, in line with our findings, an energy source switch from carbohydrates to fat in skeletal muscle. Interestingly, we could also demonstrate downregulation of the irisin-encoding gene *Fndc5* and its transcriptional regulator *Pparg1a*. The myokine irisin was shown to act on bone; however, in vitro and in vivo studies report controversial findings depending on the mouse model used, targeted cell type, and tissue context [34,35]. Still, there is a scientific consensus that circulating irisin increases browning of WAT and has a positive effect on glucose heomeostasis [36].

In accordance with our analyses, Kaspari et al. further found increased levels of *Ucp1* mRNA, the key mediator of adaptive thermogenesis in BAT, and a more multilocular distribution of brown adipocytes containing fewer lipids in hypothyroid mice suggesting a moderate activation of thermogenesis in BAT [8]. In eWAT, we observed increased glucose uptake and expression of *Glut4* and *Pck1* indicating a higher need for glucose, likely due to increased metabolic activity, as shown by upregulated adipogenic markers *Adipoq* and *Pparg2*. In female hypothyroid mice, browning of gWAT was induced due to impaired BAT function [10]; however, this was not the case in male mice of our study. In summary, we found reduced weights of eWAT and BAT, while metabolic activity of these tissues seemed to be increased in hypothyroid mice.

Interestingly, MAT volume also tended to be lower with hypothyroidism; nevertheless, the mRNA expression profile only showed a decrease in *Ppargc1a* transcript levels, the master regulator of mitochondrial biogenesis. Further studies should investigate glucose uptake, bone marrow cellular composition, and metabolic properties in greater detail to unravel how thyroid hormones might affect bone marrow adiposity and developmental fates of mesenchymal stem cells within the bone cavity. Although hypothyroidism did not alter fatty acid and triglyceride concentrations in serum, total cholesterol, HDL-cholesterol, and LDL-cholesterol levels were increased, which is consistent with the clinical situation [37]. Along with an abnormal histology, increased glucose uptake, and enhanced *Pparg2* ad *Il-1b* expression, this lipid profile points to pathological changes and malfunction of liver tissue in hypothyroid mice.

Given that several tissues can contribute to whole-body glucose alterations seen in hypothyroid animals, we subsequently treated male mice with zoledronate to target bone metabolism directly. Despite low bone turnover and a reduced uptake of glucose into bone, we did not observe effects on glucose tolerance or insulin sensitivity. Our findings are supported by several clinical studies that examined patients treated with bisphosphonates and found no side effects of bisphosphonate treatment on glucose homeostasis [17,38]. Together, these data suggest that impaired glucose tolerance in hypothyroidism is not mediated via impaired glucose uptake into bone.

Interestingly, mRNA expression of osteokines osteocalcin, sclerostin, and *Rankl* were altered in bone tissue of hypothyroid mice, but not mice treated with zoledronate, underlining a greater impact of hypothyroidism on osteoblast/osteocyte physiology than anti-resorptive treatment. Given that circulating levels of osteokines can be altered in human diseases such as obesity and type 2 diabetes mellitus [16], future studies should investigate whether these changes in osteokine expression due to hypothyroidism are more likely the cause or the result of altered whole-body glucose metabolism.

Despite its strengths, our study has potential limitations. The sex is highly relevant for thyroid disorder-induced phenotypic changes [9], and studies should be therefore repeated in female mice. Additionally, we used only drug-induced models of bone remodeling arrest whose efficiency rely on the dose, onset, and duration of treatment. Furthermore, we did not assess glucose uptake into the brain, which also has a high glucose demand and may contribute to alterations in glucose homeostasis in hypothyroidism.

Taken together, despite the close connection of bone remodeling and glucose metabolism, our data suggest that the suppression of bone turnover is not sufficient to affect whole-body glucose homeostasis in male mice.

## Data Availability

The data of this study are available from the corresponding author upon request.

## Supplementary Materials

The following supporting information can be downloaded at: https://www.mdpi.com/article/10.3390/jpm12091462/s1, Figure S1: Trabecular bone gain in mice with hypothyroidism. Bones from 16-week-old male hypothyroid (HYPO) and control (CO) mice were examined by microCT. At the spine, (A) bone mineral density (BMD), (B) trabecular separation (Tb.Sp) and (C) trabecular thickness (Tb.Th) were determined. (D) Elastic modulus (E_mod_) was determined by a compression test of the L5 vertebra. At the distal femur, (E) BMD, (F) Tb.Sp and (G) Tb.Th were assessed. (H) Cortical BMD (Ct.BMD) and (I) cortical thickness (Ct.Th) were measured at the femoral midshaft. (J) E_mod_ was determined by 3-point bending testing of femurs. (K) Representative 3D reconstructions of the trabecular compartment of the femur. Each dot indicates an individual mouse. The horizontal lines represent the mean ± 95% CI. N = 13 per group. Statistical analysis was performed by Student’s *t*-test: * *p* < 0.05; ** *p* < 0.01; *** *p* < 0.001 vs. control.; Figure S2: Mice treated with zoledronate display trabecular bone gain. Bones from 16-week-old male control mice (CO) and mice treated with zoledronate (ZOL) were examined by microCT. At the spine, (A) bone mineral density (BMD), (B) trabecular separation (Tb.Sp) and (C) trabecular thickness (Tb.Th) were determined. (D) Elastic modulus (E_mod_) was determined by a compression test of the L5 vertebra. At the distal femur, (E) BMD, (F) Tb.Sp and (G) Tb.Th were assessed. (H) Cortical BMD (Ct.BMD) and (I) cortical thickness (Ct.Th) were measured at the femoral midshaft. (J) E_mod_ was determined by 3-point bending testing of femurs. (K) Representative 3D reconstructions of the trabecular compartment of the femur. Each dot indicates an individual mouse. The horizontal lines represent the mean ± 95% CI. N = 10 per group. Statistical analysis was performed by Student’s *t*-test: * *p* < 0.05; ** *p* < 0.01; *** *p* < 0.001 vs. control; Table S1: Murine primer sequences used for quantitative real-time PCR.
