# Supplementary material for: Low Bone Turnover Due to Hypothyroidism or Anti-Resorptive Treatment Does Not Affect Whole-Body Glucose Homeostasis in Male Mice
Source: J Pers Med. 2022 Sep 6;12(9):1462. doi: 10.3390/jpm12091462 (PMC9502862; doi:10.3390/jpm12091462)
Supplement: Supplementary file 1 [file jpm-12-01462-s001.zip › Supplementary figure legends and table_Lademann et al_30-08-22.pdf]

Supplementary Figure Legends

Figure S1. Trabecular bone gain in mice with hypothyroidism. Bones from 16-week-old male hypothyroid (HYPO) and control (CO) mice were examined by microCT. At the spine, (A) bone mineral density (BMD), (B) trabecular separation (Tb.Sp) and (C) trabecular thickness (Tb.Th) were determined. (D) Elastic modulus (E_{mod}) was determined by a compression test of the L5 vertebra. At the distal femur, (E) BMD, (F) Tb.Sp and (G) Tb.Th were assessed. (H) Cortical BMD (Ct.BMD) and (I) cortical thickness (Ct.Th) were measured at the femoral midshaft. (J) Elastic modulus (E_{mod}) was determined by 3-point bending testing of femurs. (K) Representative 3D reconstructions of the trabecular compartment of the femur. Each dot indicates an individual mouse. The horizontal lines represent the mean ± 95% CI. N=13 per group. Statistical analysis was performed by Student's *t*-test: * $p<0.05$; ** $p<0.01$; *** $p<0.001$ vs. control.

Figure S2. Mice treated with zoledronate display trabecular bone gain. Bones from 16-week-old male control mice (CO) and mice treated with zoledronate (ZOL) were examined by microCT. At the spine, (A) bone mineral density (BMD), (B) trabecular separation (Tb.Sp) and (C) trabecular thickness (Tb.Th) were determined. (D) Elastic modulus (E_{mod}) was determined by a compression test of the L5 vertebra. At the distal femur, (E) BMD, (F) Tb.Sp and (G) Tb.Th were assessed. (H) Cortical BMD (Ct.BMD) and (I) cortical thickness (Ct.Th) were measured at the femoral midshaft. (J) Elastic modulus (E_{mod}) was determined by 3-point bending testing of femurs. K) Representative 3D reconstructions of the trabecular compartment of the femur. Each dot indicates an individual mouse. The horizontal lines represent the mean ± 95% CI. N=10 per group. Statistical analysis was performed by Student's *t*-test: * $p<0.05$; ** $p<0.01$; *** $p<0.001$ vs. control.

Supplemental Tables

Supplementary Table S1. Murine primer sequences used for quantitative real-time PCR.

**Target gene**	**Primer sequences 5'→3'**	
	**Sense**	**Antisense**
AdipoQ	GCACTGGCAAGTTCTACTGCAA	GTAGGTGAAGAGAACGGCCTTGT
Alp	CTACTTGTGTGGCGTGAAGG	CTGGTGGCATCTCGTTATCC
Bglap2 (Ocn)	GCGCTCTGTCTCTCTGACCT	ACCTTATTGCCCTCCTGCTT
Col1a	ACTGTCCCAACCCCCAAAG	CGTATTCTTCCGGGCAGAAA
Dio2	TGTGTCTGGAACAGCTTCCTC	ACACTGGAATTGGGAGCATC
Fndc5	AGCCCCTGTGAACGTGAC	CGCACATCCTTCTTCTGCTG
G6pd	TGCATTCCTGTATGGTAGTGG	GAATGAGAGCTCTTGGCTGG
Glut1	GCAGTTCGGCTATAACACTGG	AGAGACCAAAGCGTGGTGAG
Glut4	GGGTCCTTACGTCTTCCTTCT	CCTCTGGTTTCAGGCACTTT
Hprt1	GAGGAGTCCTGTTGATGTTGCCAG	GGCTGGCCTATAGGCTCATAGTGC

IL-1b	ACAAGGAGAACCAAGCAACG	GCCGTCTTTCATTACACAGG
IL-6	ACTTCCATCCAGTTGCCTTC	ATTTCCACGATTTCCCAGAG
Klf9	GGCTGTGGGAAAGTCTATGG	AAGGGCCGTTCACCTGTATG
Myod1	CGGGACATAGACTTGACAGGC	TCGAAACACGGGTCATCATAGA
Myog	GAGACATCCCCCTATTTCTACCA	GCTCAGTCCGCTCATAGCC
Opg	CCTTGCCCTGACCACTCTTA	ACACTGGGCTGCAATACACA
Pepck	ATGGGGTGTTTGTAGGAGCA	GAAGAGGGTTTGGGTTGTAGA
Pparg	TGAAACTCTGGGAGATTCTCCTG	CCATGGTAATTTCTTGTGAAGTGC
Ppargc1a	CCCTGCCATTGTTAAGACC	GCGTGCATCCGCTTGTG
Prdm16	CAGCACGGTGAAGCCATTC	GCGTGCATCCGCTTGTG
Rankl	CCAAGATCTCTAACATGACG	CACCATCAGCTGAAGATAGT
Runx2	AAATGCCTCCGCTGTTATGAA	GCTCCGGCCCACAAATCT
Sost	CGGAGAATGGAGGCAGAC	GTCAGGAAGCGGGTGTAGTG
Spp1	TGAAAGTGACTGATTCTGGCA	GGACGATTGGAGTGAAAGTGT
Tbp	GAAGCTGCGGTACAATTCCAG	CCCCTTGTACCCTTCACCAAT
Tnfα	GCTGAGCTCAAACCCTGGTA	CGGACTCCGCAAAGTCTAAG
Ucp1	TGCCTGGCAGATATCATCAC	CAGACCGCTGTACAGTTTCG
βAct	GATCTGGCACCACACCTTCT	GGGGTGTTGAAGGTCTCAAA

Adipoq = Adiponectin, Alp = alkaline phosphatase, Bglap2 = Osteocalcin, Col1a = Collagen Type I Alpha 1 Chain, Dio2 = Deiodinase 2, Fndc5 = Fibronectin Type III Domain Containing 5, G6pd = Glucose-6-Phosphate Dehydrogenase, Glut1 (Slc2a1) = Glucose Transporter Type 1, Glut4 (Slc2a4) = Glucose Transporter Type 4, Hprt1 = Hypoxanthine Phosphoribosyltransferase 1, IL-1b = Interleukin 1 Beta, IL-6 = Interleukin 6, Klf9 = Kruppel Like Factor 9, Myod1 = Myogenic Differentiation 1, Myog = Myogenin, Opg = osteoprotegerin, Pck1 = Phosphoenolpyruvate Carboxykinase 1, Pparg = Peroxisome Proliferator Activated Receptor Gamma, Ppargc1a = PPARG Coactivator 1 Alpha, Prdm16 = PR/SET Domain 16, Rankl (Tnfsf11) = Receptor Activator Of Nuclear Factor Kappa B Ligand, Runx2 = Runt-Related Transcription Factor 2, Sost = sclerostin, Spp1 (Opn) = Secreted Phosphoprotein 1/Osteopontin, Tbp = TATA-Box Binding Protein, Tnfα = Tumor Necrosis Factor alpha, Ucp1 = Uncoupling protein 1
