# Supplementary figures and images for: Low Bone Turnover Due to Hypothyroidism or Anti-Resorptive Treatment Does Not Affect Whole-Body Glucose Homeostasis in Male Mice
Source: J Pers Med. 2022 Sep 6;12(9):1462. doi: 10.3390/jpm12091462 (PMC9502862; doi:10.3390/jpm12091462)

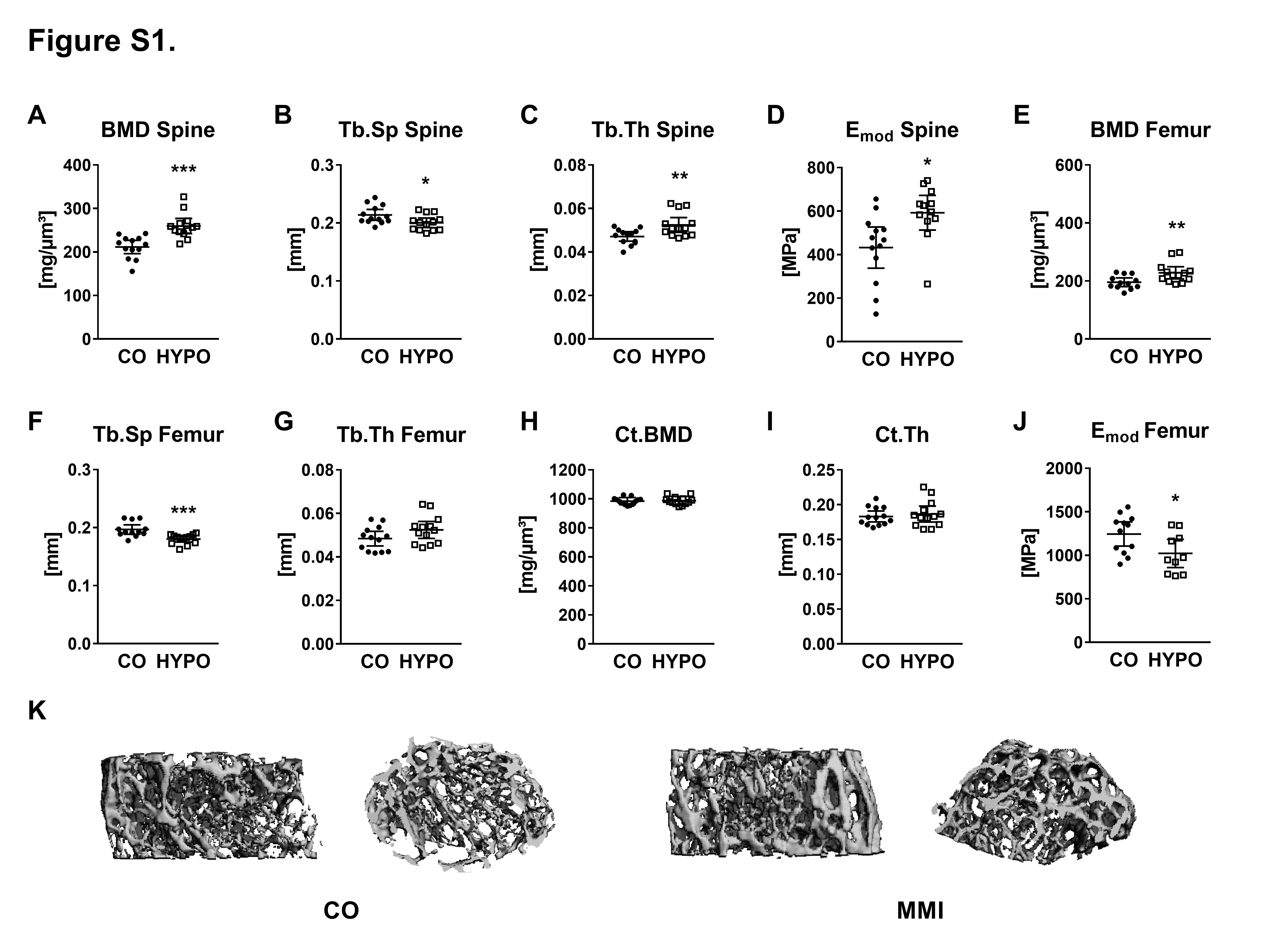

Supplement: Supplementary file 1 [file jpm-12-01462-s001.zip › Figure S1..tif]

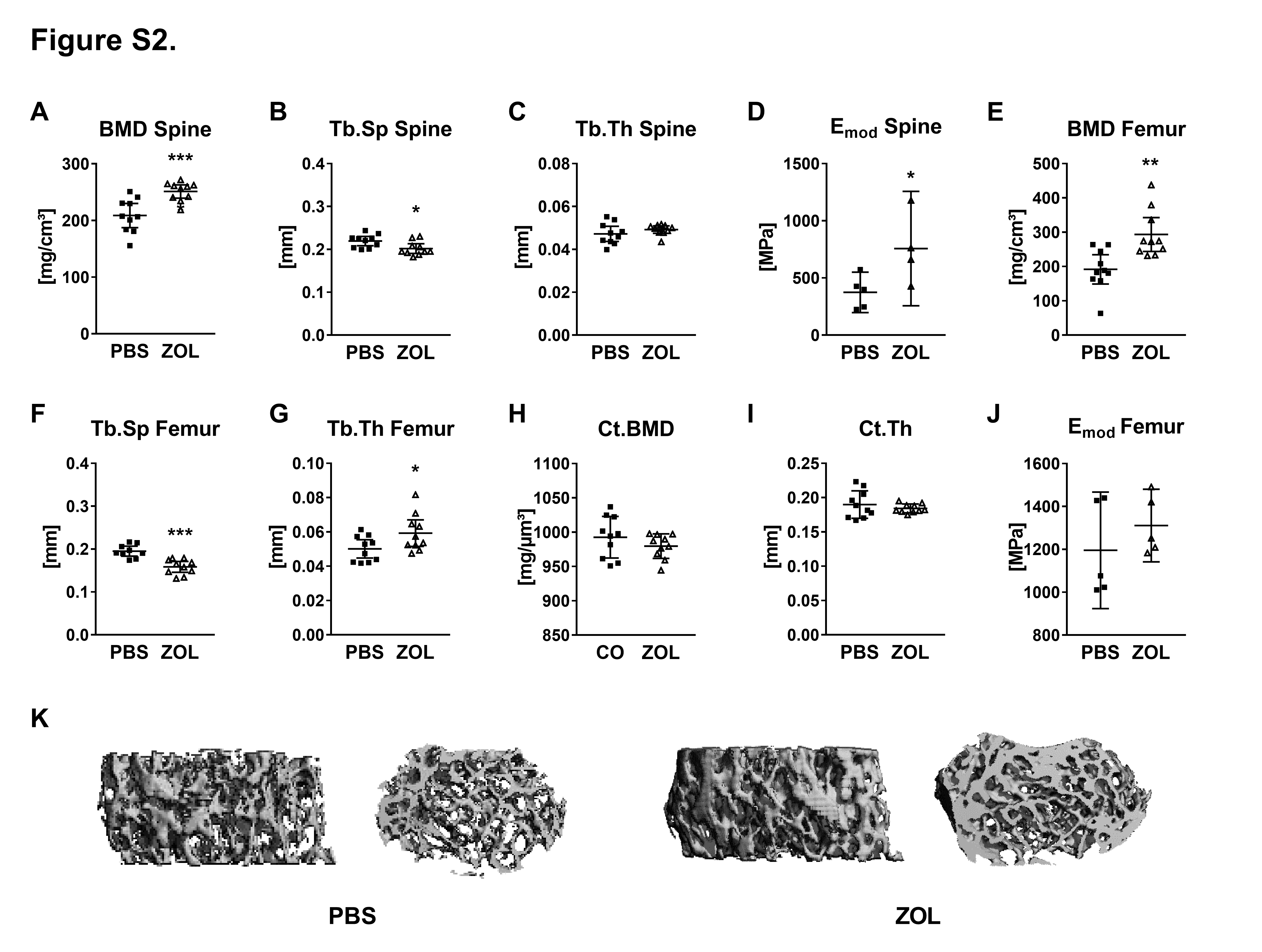

Supplement: Supplementary file 1 [file jpm-12-01462-s001.zip › Figure S2..tif]
